# The clinical stages of psychosis among violent and non-violent adult prisoners in Australia

**DOI:** 10.1192/j.eurpsy.2023.929

**Published:** 2023-07-19

**Authors:** N. Yee

**Affiliations:** Justice Health NSW | University of New South Wales (UNSW), Sydney, Australia

## Abstract

**Introduction:**

Past research examining the relationship between psychosis and criminality has typically focused on chronic schizophrenia and violence. However, contact with the criminal justice system is not constrained to the most unwell or most violent. The present study is novel as it examines the different clinical stages of psychosis, from the at-risk mental states (ARMS)/Ultra-High Risk (UHR) to the early and chronic psychotic illness phase, across the entire spectrum of criminal offending.

**Objectives:**

The main study objective is to establish the prevalence of the clinical stages of psychosis among adults entering custody and to examine the sociodemographic and forensic characteristics associated with the different stages of psychosis. A further aim is to examine whether psychosis-spectrum prisoners differ from non-psychotic prison controls across these characteristics.

**Methods:**

Participants consist of unselected 291 adult male and female prisoners entering the largest maximum security reception centres in New South Wales (NSW), Australia. They completed a range of semi-structured questionnaires and adapted mental health screening measures. The Comprehensive Assessment of At Risk Mental States (CAARMS; Yung et al., 2005) was used to ascertain whether participants met the Ultra High Risk (UHR), First Episode of Psychosis (FEP) or Established Psychosis (EP) criteria.

**Results:**

Participants were 34.25 years old (SD = 10.69) on average and men were significantly older than women (*p* = 0.035). Among prisoners with a psychosis-spectrum illness (*n* = 121), the prevalence of UHR was 24%, First Episode Psychosis (FEP) was 6% and established psychosis was 11%. Compared to controls, psychosis spectrum prisoners were found to have higher levels of social disadvantage, psychiatric comorbidities and multiple incarceration episodes. However, psychosis was not associated with a greater risk of violent offending. Implications on the complex illness burden associated with psychosis and the need for early identification and intervention across forensic mental health services will be further discussed.

**Conclusions:**

This study is novel as it examines the full spectrum of psychotic illness across the entire spectrum of criminal offending. The findings support the notion that risk of criminal justice contact and complex illness burden exist across the different clinical stages of psychosis, from the UHR to the early FEP and chronic psychosis stages, for both violent and non-violent offending. Early intervention services must consider how to more effectively identify and intervene to reduce the risk of criminal justice system contact among mentally ill individuals.

**Disclosure of Interest:**

None Declared

